# Dendritic Cells and Hepatocytes Use Distinct Pathways to Process Protective Antigen from *Plasmodium in vivo*

**DOI:** 10.1371/journal.ppat.1001318

**Published:** 2011-03-17

**Authors:** Ian A. Cockburn, Sze-Wah Tse, Andrea J. Radtke, Prakash Srinivasan, Yun-Chi Chen, Photini Sinnis, Fidel Zavala

**Affiliations:** 1 Johns Hopkins Malaria Research Institute and Department of Molecular Microbiology and Immunology, Johns Hopkins Bloomberg School of Public Health, Johns Hopkins University, Baltimore, Maryland, United States of America; 2 Department of Medical Parasitology, New York University School of Medicine, New York, New York, United States of America; Queensland Institute of Medical Research, Australia

## Abstract

Malaria-protective CD8+ T cells specific for the circumsporozoite (CS) protein are primed by dendritic cells (DCs) after sporozoite injection by infected mosquitoes. The primed cells then eliminate parasite liver stages after recognizing the CS epitopes presented by hepatocytes. To define the *in vivo* processing of CS by DCs and hepatocytes, we generated parasites carrying a mutant CS protein containing the H-2K^b^ epitope SIINFEKL, and evaluated the T cell response using transgenic and mutant mice. We determined that in both DCs and hepatocytes CS epitopes must reach the cytosol and use the TAP transporters to access the ER. Furthermore, we used endosomal mutant (3d) and cytochrome *c* treated mice to address the role of cross-presentation in the priming and effector phases of the T cell response. We determined that in DCs, CS is cross-presented via endosomes while, conversely, in hepatocytes protein must be secreted directly into the cytosol. This suggests that the main targets of protective CD8+ T cells are parasite proteins exported to the hepatocyte cytosol. Surprisingly, however, secretion of the CS protein into hepatocytes was not dependent upon parasite-export (Pexel/VTS) motifs in this protein. Together, these results indicate that the presentation of epitopes to CD8+ T cells follows distinct pathways in DCs when the immune response is induced and in hepatocytes during the effector phase.

## Author Summary

Malaria causes the deaths of 0.5–2 million people each year, mainly in Africa. A safe and effective vaccine is likely needed for the control or eradication of this disease. Immunization by irradiated malaria-infected mosquitoes has been shown to protect people against malaria. Irradiated parasites do not divide and cause infection but are capable of activating specialized killer cells called CD8+ T cells, which can protect against live parasites. Because vaccinating people with irradiated mosquitoes is not practical, we wanted to understand which parasite molecules are targeted by CD8+ T cells. These molecules may then be formulated into a safe and effective vaccine. CD8+ T cells do not automatically recognize every parasite molecule, but instead fragments of parasite proteins must be displayed on the surface of infected cells to be seen by CD8+ T cells. Our data show that CD8+ T cells recognize parasite proteins secreted by the parasite into the infected cell. This suggests that such proteins could be important components of malaria vaccines.

## Introduction

Immunization with irradiated *Plasmodium* sporozoites to induce sterile protection against live parasite challenge is a powerful model for malaria vaccination [1]. Protective immunity is mediated in part by CD8+ T cells specific for the circumsporozoite (CS) protein of *Plasmodium* [2], [3]. *Plasmodium* specific CD8+ T cells have been shown to be primed by dendritic cells (DCs) [4], [5], [6], [7]. In particular, we have found that after sporozoite inoculation into the dermis by infected mosquitoes, antigen is presented by DCs in the skin-draining lymph node to initiate the CD8+ T cell response [4]. Primed CD8+ T cells then exit the priming site and migrate to the liver where they can eliminate infection after recognizing antigen presented by hepatocytes [4]. Thus CD8+ T cell mediated immunity requires antigen presentation by two different cell types – DCs and hepatocytes. Determining how DCs and hepatocytes process and present *Plasmodium* antigens is essential for the rational identification of vaccine candidates. Since immunization with irradiated sporozoites represents the gold standard for malaria vaccination it is important to know which sporozoite antigens are presented by DCs. Perhaps more vital still, is to understand which molecules are presented by hepatocytes, as only those molecules presented to effector cells can be the targets of protective immunity.

Microbial and tumor epitopes presented by MHC class I usually derive from proteins in the cytosol that are proteolytically cleaved into small peptides by the proteasome. These peptides are translocated from the cytosol into the ER by the TAP transporter for loading onto class I MHC molecules, which then traffic towards the cell surface (reviewed in [8]). Many parasites, however, reside within a parasitophorous vacuole (PV) and their proteins are not necessarily secreted into the host cytosol. The processing and presentation of intracellular parasite antigens is therefore complex and still poorly understood. *Toxoplasma gondii* antigens have been reported to reach the cytosol for class I processing via fusion of the PV and the host ER; from the host ER antigens may be retrotranslocated into the host cytosol for processing [9]. *Leishmania major* antigens may bypass the host cytosol altogether as antigen presentation appears to be TAP independent. Instead it is believed that *L. major*-derived peptides are directly loaded onto MHC Class I in the phagolysosome [10].

The *in vivo* processing of *Plasmodium* sporozoite or liver stage antigens has not been studied. Unlike *Toxoplasma* or *Leishmania*, *Plasmodium* does not infect professional APCs and it is not known how DCs acquire sporozoite antigen. Likewise, the presentation of antigens by hepatocytes to effector cells is also poorly understood. *In-vitro* evidence suggests that hepatocytes are capable of presenting *Plasmodium* antigen and that this may be proteasome dependent [11], requiring the export of parasite antigen to the hepatocyte cytosol by unknown mechanisms. It has been proposed that Pexel/VTS motifs, known to be important for the export of proteins out of the PV in *Plasmodium* blood stages [12], [13], could also be involved in the transport of liver stage antigens to the hepatocyte cytosol for processing and presentation by class I MHC [14].

In this study we aimed to identify key cellular and molecular features of the antigen processing pathways employed by DCs and hepatocytes. We aimed to determine if *Plasmodium* CS processing requires the use of the cytoplasmic TAP dependent pathway to transport the processed epitope from the cytosol to the ER and allow binding of the peptide to class I MHC. In addition, we wanted to investigate whether the CS antigen is phagocytosed by presenting cells or if it is directly deposited or secreted into the cytosol of DCs or hepatocytes. To address these questions we generated *P. berghei* parasites that express a mutant CS protein containing the model SIINFEKL H-2K^b^ restricted epitope. Using this parasite in conjunction with knockout and mutant mice we have been able to generate the clearest picture to date of the processing of the CS protein from both sporozoite and liver stages.

## Results

### Generation of *P. berghei* CS^5M^ parasites expressing SIINFEKL in the CS protein

**Figure 1:**
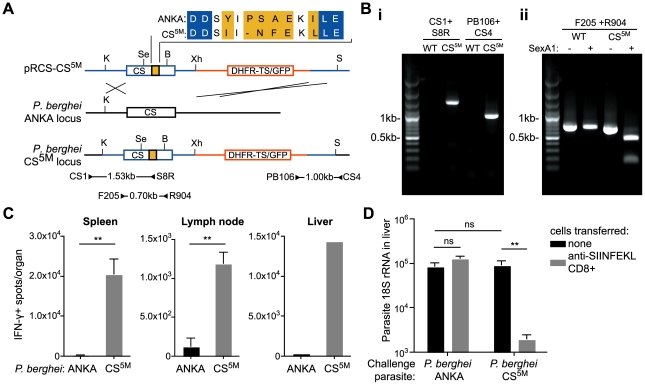
Generation of *P. berghei* CS^5M^ parasites. A. Scheme of the strategy used for gene targeting of the replacement CS^5M^ molecule. Location of primers used for PCR verification of recombination is given below (primer sequences given in Table S1). Restriction sites are K – KpnI; Se – SexAI; Bs – BsmF1; X – XhoI; S – SacI. B. Verification of clones – i. genomic DNA from cloned parasites was amplified with the primers CS1 and S8R (giving a 1526 bp product) to verify recombination at the 5′ end, and the primers CS4 and PB106 (giving a 1001 bp product) to verify recombination at the 3′ end, genomic DNA from *P. berghei* ANKA was used as a control. ii. To verify that the parasite population was clonal, genomic DNA was amplified within the CS sequence with the primers F205 and R904 to give a 699 bp product. The PCR product was then digested with SexA1, which cuts in the *P. berghei* CS^5M^ product, but not the *P. berghei* ANKA product, to yield fragments of 510 and 186 bp. C. C57Bl/6 mice were immunized i.d. in the right ear with 5×10^4^ irradiated *P. berghei* ANKA or *P. berghei* CS^5M^ parasites. 10 days later the SIINFEKL-specific immune response in the spleen, draining lymph nodes and liver (pooled) was determined by ELISPOT (mean ± SEM; n = 3, data from one of 3 similar experiments; **  =  P<0.01). D. C57Bl/6 mice received 2×10^6^ SIINFEKL-specific effector CD8+ T cells 3 hours prior to challenge with 5×10^3^ *P. berghei* ANKA or *P. berghei* CS^5M^ sporozoites (grey bars); control mice did not receive effector cells (black bars). 40 hours later livers were taken and parasite rRNA concentration determined by real-time PCR (mean ± SEM; n = 4, data from one of 2 similar experiments, ns  =  not significant).

A major obstacle to determining how *Plasmodium* antigens are presented to T cells is the lack of defined H-2^b^ restricted epitopes which severely limits *in vivo* studies, as many transgenic mice, which are critical to study basic aspects of immunology, are generated on a C57Bl/6 (H-2^b^) background. To overcome this, we generated *P. berghei* CS^5M^ parasites in which the endogenous *CS* gene was replaced with a modified *CS* gene carrying 5 mutations that changed the natural H-2K^d^ restricted epitope SYIPSAEKI to SIINFEKL, an H-2K^b^ restricted epitope (Figure 1A and B). *P. berghei* CS^5M^ parasites were apparently normal as they infected mosquitoes and mice similarly to parental *P. berghei* ANKA (Figure S1). Most importantly *P. berghei* CS^5M^ parasites stimulated a robust SIINFEKL specific response in C57Bl/6 mice upon immunization (Figure 1C), and activated SIINFEKL-specific CD8+ T cells from previously generated TCR transgenic mice [15] were able to eliminate the liver stages of *P. berghei* CS^5M^ (Figure 1D).

It is important to emphasize that our approach differs significantly from the more common strategy of inserting an entire foreign gene into a parasite and then tracking the immune responses to the foreign molecule. In the *P. berghei* CS^5M^ parasite SIINFEKL is inserted in place of a well-defined natural epitope, leaving intact the neighboring residues to ensure correct proteasomal processing, thus the model epitope is presented exactly as the natural CS epitope. This makes the *P. berghei* CS^5M^ parasite an excellent system in which to study antigen processing and presentation. Moreover, we anticipate that *P. berghei* CS^5M^ will be a powerful tool for use in future studies of antigen specific immune responses to malaria sporozoites.

### The presentation of sporozoite antigen by DCs is TAP dependent

**Figure 2:**
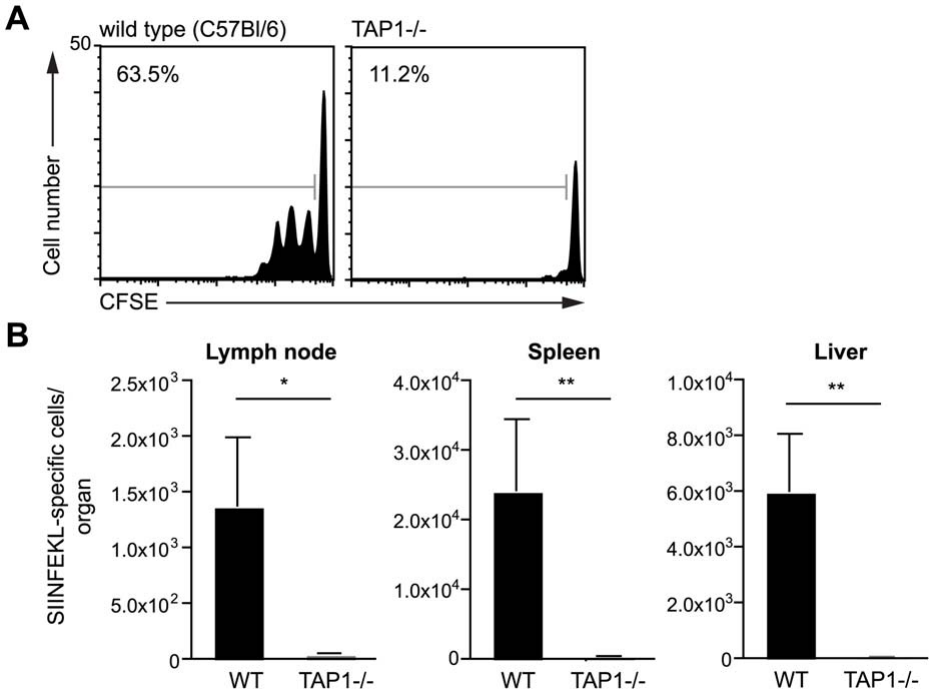
Antigen presentation by DCs is TAP dependent. A. CFSE profiles of SIINFEKL specific transgenic cells after incubation with dLN DCs isolated from C57Bl/6 (wild type) or TAP-1 deficient animals 2 days after immunization with 5×10^4^ *P. berghei* CS^5M^ sporozoites/ear. Data are based on pooled DCs from 6–8 mice per group; values at top left are the percent of cells that have divided. B. TAP1-/- and C57Bl/6 (wild type) mice received 2×10^3^ naïve SIINFEKL-specific CD8+ T cells prior to being fed on by 10–20 *P. berghei* CS^5M^ infected mosquitoes. 10 days later the mice were sacrificed and the expansion of SIINFEKL-specific cells in the spleen and liver determined. Data are pooled from 2 similar experiments (mean ± SEM; n = 6).

We initiated our studies on the presentation of *Plasmodium* antigen by investigating whether DCs present irradiated sporozoite antigen via the canonical TAP dependent pathway. Wild type and TAP-1 deficient mice [16] were immunized intra-dermally in the ear with sporozoites and 2 days later CD11c+ DCs were isolated from the draining lymph nodes. To assess antigen presentation the DCs were co-cultured with CFSE-labeled SIINFEKL specific transgenic cells. Antigen presentation was quantified by measuring the expansion of the transgenic cell population 3 days after immunization. While DCs isolated from wild type animals induced extensive proliferation of the SIINFEKL specific cells, DCs from immunized TAP-1 deficient animals were unable to induce proliferation (Figure 2A). The failure of TAP-1 deficient DCs to induce proliferation could only be due to a processing defect as TAP-1 deficient DCs pulsed with exogenous SIINFEKL peptide were fully capable of inducing antigen specific T cell proliferation (Figure S2).

To determine if TAP-1 is required *in vivo* after immunization via the natural route of infection, wild-type and TAP-1 deficient animals that had received SIINFEKL specific TCR transgenic CD8+ T cells were immunized by the bites of irradiated mosquitoes infected with *P. berghei* CS^5M^ parasites. We observed a robust antigen specific CD8+ T cell response after immunization of wild type mice; however, immunized TAP-1 deficient animals failed to mount a significant CD8+ T cell response in either the draining LN, spleen or liver (Figure 2B). Together these data indicate that the presentation of the CS protein by DCs is strictly TAP dependent.

### Sporozoite antigen presentation by DCs occurs via an endosome-to-cytosol pathway

Given that the priming of sporozoite specific T cells is TAP dependent, the CS protein must reach the cytosol of the DC for antigen processing. Since *Plasmodium* parasites have not been observed to productively infect DCs [17], [18] it is not obvious how sporozoite antigen accesses the DC cytosol. One possibility is that CS antigen from sporozoites is cross-presented via an endosome-to-cytosol pathway in which sporozoite antigen is phagocytosed and then retrotranslocated into the cytosol [19]. Alternatively, CS may be deposited in DCs during the process of cell traversal - a process in which sporozoites pass through the cytosol of cells, without forming a vacuole around themselves [20], [21], [22].

**Figure 3:**
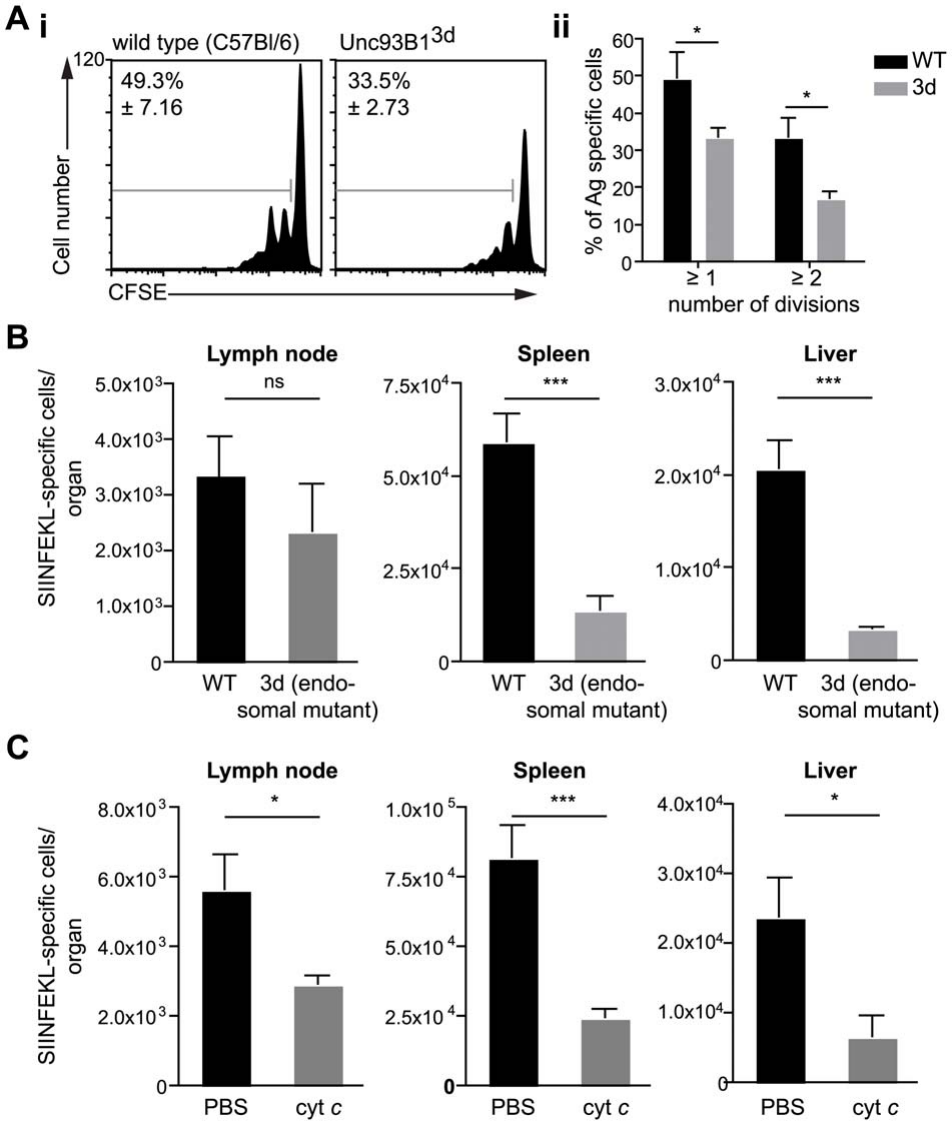
Antigen presentation by DCs occurs via the endosome-to-cytosol pathway. A. i. DCs were purified from the ear draining LN of C57Bl/6 (wild type) or Unc93B1^3d^ animals 2 days after immunization with 5×10^4^ *P. berghei* CS^5M^ sporozoites/ear and incubated with CFSE labeled SIINFEKL specific trangenic cells. i Representative CFSE profiles of the transgenic cells 3 days after immunization values at top left are the percent of cells that have divided (mean ± SEM). ii. Mean number of cells that had divided at least one or twice in 3 independent experiments after incubation with DCs from wild type (black bars) or 3d mice (gray bars) (mean ± SEM; *  =  P<0.05; for each group in each experiment pooled DCs from 6–8 immunized animals were used). B. 3d (endosomal mutant) and C57Bl/6 mice received 2×10^3^ naïve SIINFEKL-specific cells prior to being fed on by 10–20 *P. berghei* CS^5M^ infected mosquitoes. 10 days later the mice were sacrificed and the expansion of SIINFEKL-specific cells in the spleen and liver determined. Data are pooled from 2 similar experiments (mean ± SEM; n = 6; ***  =  P<0.001). C. C57Bl/6 mice received 2×10^3^ naïve SIINFEKL-specific cells prior to being fed on by 10–20 *P. berghei* CS^5M^ infected mosquitoes. Treated mice received 15 mg of horse cyt *c* (Sigma) for 3 days starting on the day before immunization, control mice received vehicle alone (PBS). 10 days later the mice were sacrificed and the expansion of SIINFEKL-specific cells in the spleen and liver determined. Data are pooled from 2 similar experiments (mean ± SEM; n = 6).

To distinguish between these possibilities we evaluated the induction of CD8+ T cell responses in animals which have a single-point mutation in the molecule Unc93B1 (3d mice). This mutation causes several impairments to endosome function including defects in signaling via the endosomal TLRs and in cross presentation [23]. We reasoned that if there were defects in T cell priming in these animals it would strongly indicate a role for endosomes in antigen processing by DCs. We found that DCs isolated from immunized 3d mice were less capable of priming SIINFEKL specific T cells *in vitro* compared to wild type controls (Figure 3A). This defect appears to be in the processing of antigen, as exogenous peptide is efficiently presented by DCs from 3d mice (Figure S2). Nonetheless, *ex vivo* antigen presentation assays provide only a snapshot of sporozoite antigen presentation at a single time point whereas we have recently shown that prolonged antigen presentation is required for full T cell priming [24]. Thus we assessed T cell priming *in vivo* after immunization by mosquito bites. We found that the difference observed in *ex vivo* experiments was amplified *in vivo* as 3d mice had severely decreased SIINFEKL specific responses in the spleen and liver compared to wild type mice (Figure 3B).

The role of endosomes in the presentation of sporozoite antigen by DCs was further confirmed in experiments in which cross-presenting DCs subsets were depleted *in vivo* by treatment with cytochrome *c* (cyt *c*; Figure S3) [25], [26], [27]. Upon taking up cyt *c* cross-presenting DCs retrotranslocate it into the cytosol where it can induce apoptosis. In contrast non cross-presenting cell subsets are unaffected as they break down any cyt *c* that has been taken up in lysosomes. In agreement with the data from 3d mice we found significant reductions in the priming of SIINFEKL specific T cells in cyt *c* treated animals after immunization via mosquito bites (Figure 3C). Together these data demonstrate that the majority of sporozoite antigen is probably processed via the endosome-to-cytosol pathway.

### Opsonization of parasites inhibits their presentation by DCs

**Figure 4:**
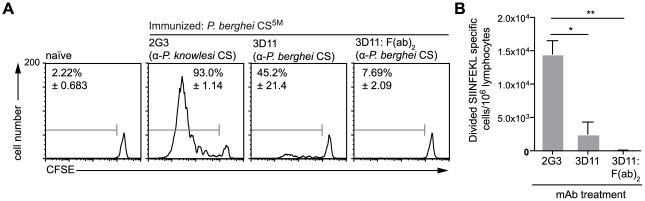
Antigen presentation by DCs is inhibited by opsonization. Mice received 5×10^5^ CFSE labeled SIINFEKL-specific cells one day prior to immunization i.d. with 5×10^4^ *P. berghei* CS^5M^ parasites in the right ear that had either been treated for 20 minutes with 100 µg/ml of either anti-*P. knowlesi* CS (2G3), anti-*P. berghei* CS (3D11) or F(ab′)_2_ fragments prepared from the 3D11 antibody. Three days later the mice were sacrificed and ear draining lymph nodes taken. A. Representative CFSE profiles of the SIINFEKL-specific population, values are the mean % of cells that had proliferated ± SEM in one of 4 similar experiments. B. The size of the expansion of the transferred SIINFEKL-specific cells (mean ± SEM n = 3; representative of 4 similar experiments).

Given that the presentation of sporozoite antigen by DCs occurs via the endosome, we hypothesized that opsonization of parasites might enhance the priming of CD8+ T cells [28], [29]. Accordingly we incubated parasites with the anti-CS mAb 3D11 [30] prior to immunization. Unexpectedly, we found that opsonized parasites induced much reduced proliferation of CD8+ T cells compared to sporozoites treated with irrelevant antibody [31] (Figure 4). This intriguing result indicates that opsonization inhibits rather than potentiates the delivery of sporozoite derived CS protein to the DC class I processing pathway. This surprising result is not completely unprecedented – opsonized *T. gondii* parasites appear to be taken up by DCs via complement and Fc receptors and directed away from the cross presenting pathway and towards break down by lysosomes [9]. To determine if this occurs after opsonization of *Plasmodium* sporozoites we also treated sporozoites with F(ab′)_2_ fragments of the 3D11 mAb which cannot be recognized by Fc receptors and do not efficiently fix complement. However 3D11 F(ab′)_2_ fragments were as efficient as intact antibody at inhibiting T cell priming. Thus it may be that opsonization (and F(ab′)_2_ treatment) affect T cell priming by immobilizing parasites [32] and thus interfering with a number of processes which may be important for T cell priming. These include parasite migration to the skin draining lymph nodes, invasion of cells in the skin and the shedding of antigen from the sporozoite surface [4], [17], [33].

### Hepatocytes present *Plasmodium* antigens that are directly secreted into the cytosol

Because effector cells must kill infected hepatocytes, it is also required that hepatocytes present processed antigen to CD8+ T cells. Therefore, in addition to DCs, we were also interested in determining how hepatocytes process antigen for presentation to effector cells. To determine if antigen is processed by hepatocytes via the same endosome-to-cytosol pathway employed by DCs, activated SIINFEKL specific CD8+ T cells were transferred to TAP-1 deficient, 3d and cyt *c* treated mice that were subsequently infected with *P. berghei* CS^5M^ parasites. The read-out for epitope presentation is T-cell mediated inhibition of liver stage development i.e. if the epitope is presented, activated CD8+ T cells will recognize it and will eliminate liver stage parasites. We also tried to visualize antigen presentation by immuno-fluorescence with the mAb 25-D1.16 which recognizes K^b^-SIINFEKL complexes [34]; however, in common with other researchers we found that this technique was not sensitive enough to detect epitopes on the surface of parasite infected cells [35].

**Figure 5:**
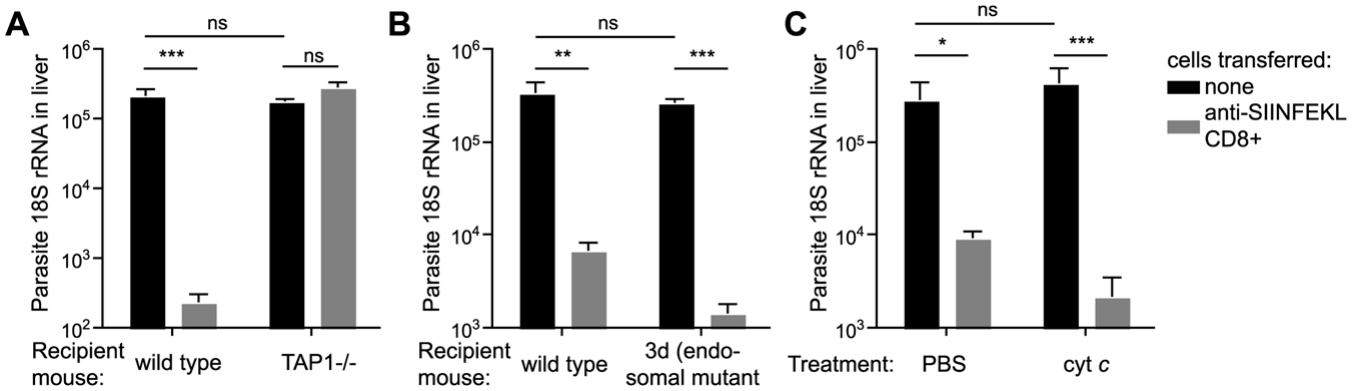
Antigen is directly presented to effector cells by hepatocytes. A. C57Bl/6 or TAP1-/- mice received 2×10^6^ SIINFEKL-specific effector CD8+ T cells 3 hours prior to challenge with 5×10^3^ *P. berghei* CS^5M^ sporozoites (grey bars); control mice did not receive effector cells (black bars). 40 hours later livers were taken and parasite rRNA concentration determined by real-time PCR (mean ± SEM; n = 4, data from one of 2 similar experiments). B. C57Bl/6 or 3d mice received 2×10^6^ SIINFEKL specific effector CD8+ T cells 3 hours prior to challenge with 5×10^3^ *P. berghei* CS^5M^ sporozoites (grey bars); control mice did not receive effector cells (black bars). 40 hours later livers were taken and parasite rRNA concentration determined by real-time PCR (mean ± SEM; n = 5, data from one of 2 similar experiments). C. Cyt *c* or PBS treated C57Bl/6 mice received 2×10^6^ SIINFEKL specific effector CD8+ T cells 3 hours prior to challenge with 5×10^3^ *P. berghei* CS^5M^ sporozoites (grey bars); control mice did not receive effector cells (black bars). Treated mice received 15 mg cyt *c* for 3 days starting the day before challenge. 40 hours after challenge livers were taken and parasite rRNA concentration determined by real-time PCR (mean ± SEM; n = 4).

Using our *in vivo* functional assay we found that effector CD8+ T cells had no inhibitory effect on parasite development in the livers of TAP-1 deficient animals while they were fully capable of eliminating parasites in wild type mice (Figure 5A), clearly indicating that in hepatocytes, as in DCs, CS must reach the cytosol for antigen processing. However, in sharp contrast to DCs, we found that hepatocytes do not process antigen via endosomes since effector CD8+ T cells were capable of efficiently eliminating parasites from the livers of 3d or cyt *c* treated mice (Figure 5B and C). Thus hepatocytes unlike DCs do not appear to process antigen by an endosome to cytosol pathway, rather, hepatocytes present antigen that has been deposited or secreted by the parasite directly into the cytosol.

### Presentation of CS by infected hepatocytes and DCs does not require functional Pexel/VTS motifs

**Figure 6:**
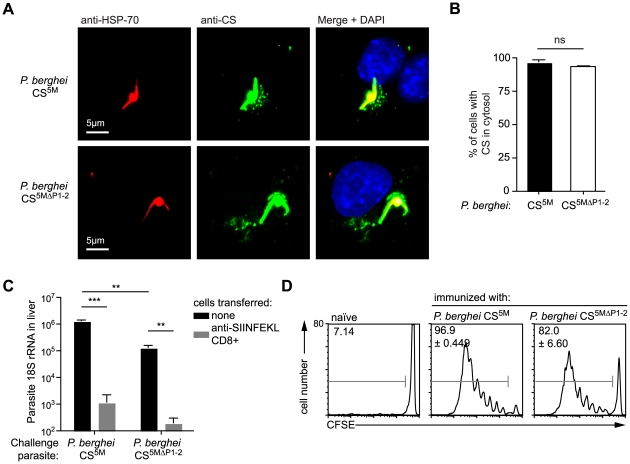
Pexel/VTS motifs are not required for the presentation of CD8+ epitopes in the CS protein. A. Fluorescence microscopy of Hepa1–6 cells 6 hours after infection with *P. berghei* CS^5M^ and *P. berghei* ^CS5MΔP1–2^. Parasites were visualized by staining with anti-*Plasmodium* HSP70 (red) and the localization of the CS protein determined by staining with the 3D11 mAb (green). B. % of parasites with CS visible in the host cell based on microscopy performed as in A, (mean ± SEM; data are based on 3 independent experiments per parasite strain with 50 parasites imaged per experiment). C. C57Bl/6 mice received 2×10^6^ SIINFEKL specific effector CD8+ T cells 3 hours prior to challenge with 5×10^3^ *P. berghei* CS^5M^ or *P. berghei* CS^5MΔP1–2^ sporozoites (grey bars); control mice did not receive effector cells (black bars). 40 hours later livers were taken and parasite rRNA concentration determined by real-time PCR (mean ± SEM; n = 4, data from one of 2 similar experiments). D. C57Bl/6 mice received 5×10^5^ CFSE labeled naïve SIINFEKL-specific cells one day prior to immunization i.d. with 5×10^4^ *P. berghei* CS^5M^ or *P. berghei* CS^5MΔP1–2^ parasites in the right ear. Three days later the mice were sacrificed and ear draining lymph nodes taken. Antigen presentation *in vivo* was inferred by determining the % of SIINFEKL-specific cells that had proliferated (mean ± SEM; n = 3, data representative of 2 similar experiments).

Our findings that antigen presentation in hepatocytes requires CS to enter the host cytosol but is independent of the endosomal pathway, raise the question as to how CS traffics to the hepatocyte cytosol. A previous report in which the 2 Pexel/VTS motifs in the N terminal domain of CS were mutated, suggested that CS export to the cytosol was eliminated in the absence of functional Pexel/VTS motifs [14]. To determine whether Pexel/VTS motifs are critical for the entry of CS into the class I processing pathway of infected hepatocytes we generated *P. berghei* CS^5M^ parasites that carried mutations in key residues of both Pexel/VTS motifs as well as the SIINFEKL epitope (*P. berghei* CS^5MΔP1–2^; Figure S4). We mutated the Pexel/VTS sequences to the sequence that was previously suggested to abolish CS export into the cytoplasm of infected hepatocytes [14]. In fact we were able to observe punctate staining of CS in the cytosol of both *P. berghei* CS^5M^ and *P. berghei* CS^5MΔP1–2^ infected Hepa1-6 cells (Figure 6A and B), and more importantly, we found that the *P. berghei* CS^5MΔP1–2^ parasites were killed as efficiently as *P. berghei* CS^5M^ by effector CD8+ T cells (Figure 6C). This indicates that Pexel/VTS motifs are not required for the entry of CS into the cytosol of hepatocytes for antigen presentation to effector CD8+ T cells. However, in agreement with the previous study we did observe that parasites with mutated Pexel/VTS motifs in the CS protein have a ∼10-fold decrease in infectivity (Figure 6C). Finally we found that DCs efficiently present the epitope from the CS protein of parasites lacking the Pexel/VTS motifs (Figure 6D). This was not entirely unexpected as our previous findings suggested that DCs likely acquire the CS antigen by phagocytosis which is unlikely to be affected by host cell targeting sequences.

## Discussion

In this study we demonstrate that the process of antigen presentation required for the priming of sporozoite specific T cells and for the elimination of liver stage parasites are distinct. The difference in antigen presentation between DCs and hepatocytes has important consequences for malaria vaccine development based on irradiated sporozoites. If other *Plasmodium* antigens are processed similarly to CS, it is likely that DCs, which acquire antigens by phagocytosis, could stimulate T cell responses to a broad range of secreted and non-secreted antigens. In contrast hepatocytes can only present antigens that are secreted into the cytosol of infected or traversed cells; these antigens are, however, the potential targets of protective immunity as they induce effector cells to eliminate liver stage parasites. Thus, irradiated sporozoites may induce a range of irrelevant as well as protective immune responses. Moreover it is possible that irradiated sporozoites will fail to induce protective responses to various liver stage antigens presented by hepatocytes, that are not expressed by sporozoites. This appears to be the case for the liver stage antigen Hep17: irradiated sporozoites do not induce detectable Hep17 specific CD8+ T cells; however, vaccine-induced T cells specific for this antigen are protective against *Plasmodium* liver stages [36].

We observed that both T cell priming and parasite elimination by T cells were strictly TAP dependent. Thus in both DCs and hepatocytes antigen must reach the cytosol for presentation. In DCs this appears to occur via an endosome-to-cytosol pathway as determined by two independent *in vivo* methodologies: the use of 3d mice and treatment of mice with cyt *c*. However, unlike the defect in TAP1 deficient mice, the reduction in T cell priming in both 3d and cyt *c* treated mice was not complete. This may indicate that a small amount of antigen is directly deposited in the cytosol of DCs by traversing sporozoites. Alternatively cross-presentation may not be fully ablated in these models. 3d mice carry a single point mutation in one molecule (Unc93B1) which may retain some residual functionality [23], while the depletion of cross-presenting DCs by cyt *c* may not be absolute, particularly in the lymph nodes. The function of Unc93B1 in antigen presentation is not clear, though it may be involved in translocating elements of the cross-presentation machinery to the endosome similar to the way it mediates the movement of TLRs to endosomes [37]. An intriguing recent study showed that 3d mice were highly susceptible to *T. gondii* infection [38]. The authors suggest that this was not due to an impairment of CD8+ T cell control of parasites as the activation of CD8+ T cells appeared normal in 3d mice – however they were only able to look at bulk T cell populations and not antigen specific cells.

Further research will be required to determine what receptors DCs use to take up sporozoites and which pattern recognition molecules interact with sporozoites to facilitate cross presentation. One unexpected finding was that opsonization of sporozoites did not enhance the presentation of the CS antigen by DCs. One hypothesis is that opsonization may immobilize parasites [32] and thus interfere with a variety of processes that may be important for T cell priming including antigen shedding, and migration to the draining lymph nodes for presentation [4], [17], [33]. Alternatively opsonization may prevent parasites from infecting cells in the skin where they could continue to provide antigen to the immune system [18], [39]. The inability of DCs to present antigen from immobilized parasites may explain why irradiated parasites are capable of inducing a protective CD8+ T cell response, but heat killed parasites are not [3], [40]. These data also have important implications for vaccine design since they imply that there would be difficulties in priming or boosting sporozoite specific CD8+ T cell responses in individuals with high anti-CS antibody titers. Thus it may be hard to induce effective CD8+ T cell responses in individuals who have already been naturally exposed to parasites or immunized with vaccines such as RTS,S that are designed to induce strong anti-sporozoite antibody responses [41].

Using the 3d and cyt *c* treated mice we showed that in contrast to T cell priming, parasite elimination was unaffected in mice with reduced capacity to cross-present antigen. This is in agreement with the findings of a previous *in vitro* study [11] which found no evidence for endosomes having a role in antigen presentation by infected cells. The previous study also showed that proteasome and Golgi inhibitors blocked antigen presentation, which is compatible with our finding that antigen presentation occurs via the classical TAP-dependent pathway [11]. Together these data suggest that cell killing occurs only after direct antigen presentation by the infected hepatocyte itself.

A key direction for future research will be to identify how antigens enter the host cell for presentation. We were unable to find a role for Pexel/VTS motifs in targeting the CS protein to the host cell cytosol as suggested by a previous study [14]. Our data are based on fluorescence microscopy 6 hours post-infection when the highest amounts of CS can be observed in the cytosol [42], [43] and, more importantly, our functional assay to measure the elimination of parasites by T cells. The fact that Pexel/VTS motifs are not required for the entry of CS to the class I processing pathway suggests that liver stage proteins may be exported to the hepatocyte by other mechanisms. In particular, it suggests that the CS protein may contain another motif that facilitates its export out of the PV into the infected host cell. Alternatively, liver-stage antigens might also be exported to the class I processing pathway if the *Plasmodium* PV can fuse with the hepatocyte ER as appears to occur in *Toxoplasma* infected DCs [9].

Together our data provide the most complete description to date of the processing of sporozoite and liver stage antigen. Using the *P. berghei* CS^5M^ parasite we have demonstrated that DCs cross-present sporozoite antigen via an endosome-to-cytosol pathway. Of most importance, we show that CS must be delivered to the hepatocyte cytosol for presentation to effector cells. If this is true for other antigens, it is likely that antigens secreted into the hepatocytes of either infected or traversed cells constitute the major targets of anti-liver stage CD8+ T cell mediated immunity. Secretion to the hepatocyte is likely a complex process given our finding that Pexel/VTS motifs are not required for the entry of CS to the class I processing pathway; however, unraveling this process will be key to the identification of vaccine candidates.

## Materials and Methods

### Ethics statement

All animal procedures were approved by the Institutional Animal Care and Use Committee of the Johns Hopkins University (Protocol Number MO09H41) following the National Institutes of Health guidelines for animal housing and care.

### Mice

5–8 week old female C57Bl/6 were purchased from NCI (Frederick, MD). TAP-1 deficient animals were purchased from Jackson (Bar Harbor, ME). Unc93B1^3d^ mice were obtained from the Mutant Mouse Resource Center (University of California, Davis, CA). OT-1 mice (carrying a transgene specific for the SIINFEKL epitope) were kindly provided by David Sacks (Laboratory of Parasitic Disease, National Institute of Allergy and Infectious Disease, Bethesda, MD).

### Parasites and transfections

*P. berghei* CS^5M^ parasites were generated by transfection of *P. berghei* ANKA with the linearized pR-CS^5M^ plasmid as previously described [44]. pR-CS^5M^ was derived from the plasmid pR-CSwt [45] as follows. A Kpn1-Xho1 fragment including the entire CS gene was excised from pR-CSwt into a pBluescript SK- (Stratagene) backbone to generate the plasmid pIC-CSwt. A SexA1 site was introduced by mutation of G to A at position 714 in the CS gene (silent in Gln238) and a BsmF1 site was introduced by a mutation of T to C at position 810 (silent in Asp270) using the QuikChange XL site directed mutagenesis kit (Stratagene). The SexA1-BsmF1 fragment was excised and replaced with a ∼100 bp insert including the SIINFEKL epitope in place of the SYIPSAEKI sequence (formed from the oligos S8ins F and S8insR; see Table S1) to generate the plasmid pIC-CS^5M^. The Kpn1-Xho1 fragment from pIC-CS^5M^ was excised and ligated into the backbone of pR-CSwt to generate the pR-CS^5M^ plasmid. pR-CS^5M^ was linearized with the enzymes Kpn1 and Sac1.

*P. berghei* CS^5MΔP1–2^ parasites were generated similarly to *P. berghei* CS^5M^ (Figure S1). The plasmid pR-CS^5MΔP1–2^ was generated as follows. Arg32 and Leu34 in the CS gene on the pIC-CS^5M^ plasmid were mutated to Alanines by using the QuikChange site directed mutagenesis kit with the primers PEXEL1 F and PEXEL1 R (see Table S1), which include a Bsm1 site. Arg66 and Leu68 were mutated similarly with the primers PEXEL2 F and PEXEL2 R that include an ApaB1 site. The resulting plasmid was designated pIC-CS^5MΔP1–2^. The Kpn1-Xho1 fragment of the pIC-CS^5MΔP1–2^ plasmid was ligated into the pR-CSwt backbone to generate the pR-CS^5MΔP1–2^ plasmid used for transfection.

### Quantification of T cell priming by DCs ex vivo

Lymph node and spleen myeloid DCs were prepared essentially as described [46]. Briefly, spleens or lymph nodes from immunized mice or naive mice were taken, chopped finely and digested with 1 mg/ml collagenase. The single cell suspension of spleen cells was then separated over a Nycodenz gradient (density, 1.075 g/ml) and the DC-rich low-density fraction was taken. To further enrich the DC population, negative selection was performed on the collected fraction using magnetic bead separation with anti-CD3, anti-GR1, anti TER119, anti-B220 and anti-Thy1.2 antibodies. Final purity of CD11c+ DC was about 70%. To assess Ag presentation *ex vivo*, splenic myeloid DCs (1×10^5^) were mixed with 5×10^4^ purified naive CFSE-labeled CD8^+^-transgenic cells in a single V-bottom well of a 96-well plate. 60–65 h later, the cells were harvested, and CFSE dilution in the transgenic cell population was used as a measure of Ag presentation.

### Quantification of T cell priming by DCs in vivo

Where possible SIINFEKL-specific T cell priming was measured after immunization by the bites of 10–20 irradiated mosquitoes. Prior to biting, a low number (2×10^3^) of CD45.1+ OT-1 cells were transferred to mice and the expansion of the CD45.1+ CD8+ (SIINFEKL-specific) population were measured by flow cytometry 10 days later to allow time for the responses to reach detectable levels. In some experiments it was necessary to perform immunizations with needle injected sporozoites (e.g. where the sporozoites were treated with antibodies prior to immunization). In these experiments 5×10^5^ congenic CD45.1+ OT-1 cells were adoptively into mice, which were immunized the following day. The cells would be labeled with 0.6 µM CFSE using the Vybrant Cell Tracker kit according to the manufacturer's instructions (Invitrogen Life Technologies), and antigen presentation was inferred from proliferation of CD45.1+ CD8+ cells in the draining lymph nodes after 3 days. Use of a high number of transgenic cells is acceptable in these experiments as we are using the cells as a readout of antigen presentation not measuring particular T cell phenotypes. ELISPOTs to measure peptide-specific IFN-γ secreting cells were performed as described [47] and used to detect endogenous SIINFEKL responses.

### Preparation of F(ab′)_2_ fragments

F(ab′)_2_ fragments from the 3D11 mAb (class: mouse IgG1) were prepared by incubation with immobilized Ficin in the presence of 4 mM cysteine according to the manufacturer's instructions (Pierce). F(ab′)_2_ fragments were isolated from intact antibody and Fc fragments by passing twice over a Protein A column. Purity of F(ab′)_2_ fragments was verified by SDS-PAGE under non-reducing conditions.

### Generation of SIINFEKL-specific effector T cells

SIINFEKL-specific effector cells were purified from mice that had received 5×10^5^ naïve CD45.1+ OT-1 cells and then been immunized with 2×10^6^ pfu VV-OVA [48]. 8–10 days later spleens were taken from the immunized mice and the lymphocytes were purified by spinning over lympholyte M (Cedarlane Laboratories). A total of 2×10^6^ effector/SIINFEKL specific CD8+ T cells were transferred to each recipient mouse.

### Quantification of parasite RNA

Quantification of liver stage parasites was performed as previously described [49]. Briefly, 40 hours after challenge, livers were excised and parasite load was determined by quantitative PCR for *P. berghei* 18S rRNA using SYBR Green (Applied Biosystems).

### Cell isolation and preparation of samples for flow cytometry

Single cell suspensions of lymphocytes were obtained by grinding spleen cells or lymph node cells between the ground ends of two microscope slides and filtering twice through 100 µm nylon mesh. Liver lymphocytes were isolated from perfused livers by grinding, filtration through a 70 µm mesh and separation over a 35% percol gradient as described [50].

### Fluorescence microscopy

Hepa1-6 cells were grown on coverslips in a 48 well plate and allowed to reach ∼80% confluence prior to infection with ∼3×10^4^ parasites. 6 hours later the slides were washed and fixed for 15 minutes with 4% formaldehyde prior to permeablilization with 100% methanol for 10 minutes. The cells were then blocked with 3% BSA for 45 minutes. The parasite cytosol was labeled with anti-*Plasmodium* HSP70 mAbs [51] followed by secondary staining with Alexa594 anti-mouse IgG (Molecular Probes). The cells were then stained with anti-*P. berghei* CS mAb (3D11) directly conjugated to FITC. Slides were mounted with ProLong antifade with DAPI (Molecular Probes). Images were acquired on a Nikon Eclipse 90i microscope with a Hamamatsu Orca-ER camera attachment using Volocity software (Perkin Elmer). Images were analyzed and assembled using ImageJ software (open source from NIH).

### Data analysis

Statistical analysis was performed using Prism 4 software (GraphPad Software), unless otherwise stated, means were compared by two-tailed Student's t tests. Analysis of all flow cytometry data was performed using FlowJo software (TreeStar).

## Supporting Information

Figure S1 Infectivity *P. berghei* CS^5M^ parasites in the mosquito and mouse. A. Salivary glands were dissected from mosquitoes 21 days after blood feeding with *P. berghei* ANKA or *P. berghei* CS^5M^ and sporozoites extracted and counted. Results are based on 3 independent feedings per group with >10 mosquitoes dissected per feeding (mean ± SEM; ns  =  not significant). B. Parasite load in the livers of mice infected with *P. berghei* ANKA or *P. berghei* CS^5M^ was assessed 40 hours after infection (mean ± SEM; n = 4, data from one of 2 similar experiments).
(0.40 MB TIF)

Figure S2 DCs from TAP-1 deficient and 3d mice can efficiently present exogenous peptide. CFSE profiles of SIINFEKL specific transgenic cells after incubation with spleen DCs isolated from C57Bl/6 (wild type), TAP-1 deficient or 3d mice that had been pulsed with 10μg/ml SIINFEKL peptide. As a control transgenic cells were also incubated with unpulsed DCs from naive wild type mice. Data are based on pooled DCs from 3 mice per group; values at top left are the percent of cells that have divided. Data is representative of two independent experiments per group.
(0.33 MB TIF)

Figure S3 Cyt *c* treatment selectively ablates cross-presenting DC populations. Mice were treated with 15 mg horse cyt *c* in PBS or PBS alone, administered i.v. and 24 hours later the number of CD4+ DCs (CD4+, CD11c+, CD8−, CD3−) CD8+ DCs (CD8+, CD11c+, CD4−, CD3−) and double negative (DN) DCs (CD4−, CD8−. CD11c+, CD3−) was assessed in the spleen (A) and skin draining LNs (B) by FACs.
(0.77 MB TIF)

Figure S4 Generation of *P. berghei* CS^5MΔP1–2^ parasites. A. Scheme of the strategy used for gene targeting of the replacement CS^5MΔP1–2^ molecule. Open reading frames are represented by boxes, untranslated regions by solid lines, plasmid vectors sequences by dotted lines. Black represents wild type genomic sequences, blue represents homologous sequences in the targeting construct, red represents sequence associated with the selectable marker, and yellow represents mutations in the CS gene. Location of primers used for PCR verification of recombination is given below (primer sequences given in Table S1). Restriction sites are K - KpnI; B - BsmI; A - ApaBI; Se - SexAI; Bs - BsmF1; X - XhoI; S - SacI. B. Verification of clones - i. genomic DNA from cloned parasites was amplified with the primers CS1 and S8R (giving a 1526 bp product) to verify recombination at the 5′ end and the primers CS4 and PB106 (giving a 1001 bp product) to verify recombination at the 3′ end, genomic DNA from *P. berghei* ANKA was used as a control. ii To verify that parasite populations was clonal genomic DNA was amplified within the CS sequence with the primers F205 and R904 to give an 699 bp product in *P. berghei* ANKA and 696 bp product in *P. berghei* CS^5MΔP1–2^ (which has one codon less). The PCR product was then digested with SexA1 which cuts in the *P. berghei* CS^5MΔP1–2^ product, but not the *P. berghei* ANKA product, to yield fragments of 510 and 186 bp. iii To verify that the parasites carried mutations in the PEXEL domains, the CS1 and S8R PCR product was digested with the enzymes BsmI which cuts in the mutated Pexel1 motif to yield 618 and 845 bp fragments and ApaBI which cuts in the mutated Pexel2 motif to yield 581 and 945 bp fragments. The PCR product from the *P. berghei* CS^5M^ parasite was used as a control.
(1.46 MB TIF)

Table S1 Oligonucleotides used in this study.
(0.05 MB DOC)
