# Supplementary figures and images for: Dendritic Cells and Hepatocytes Use Distinct Pathways to Process Protective Antigen from Plasmodium in vivo
Source: PLoS Pathog. 2011 Mar 17;7(3):e1001318. doi: 10.1371/journal.ppat.1001318 (PMC3060173; doi:10.1371/journal.ppat.1001318)

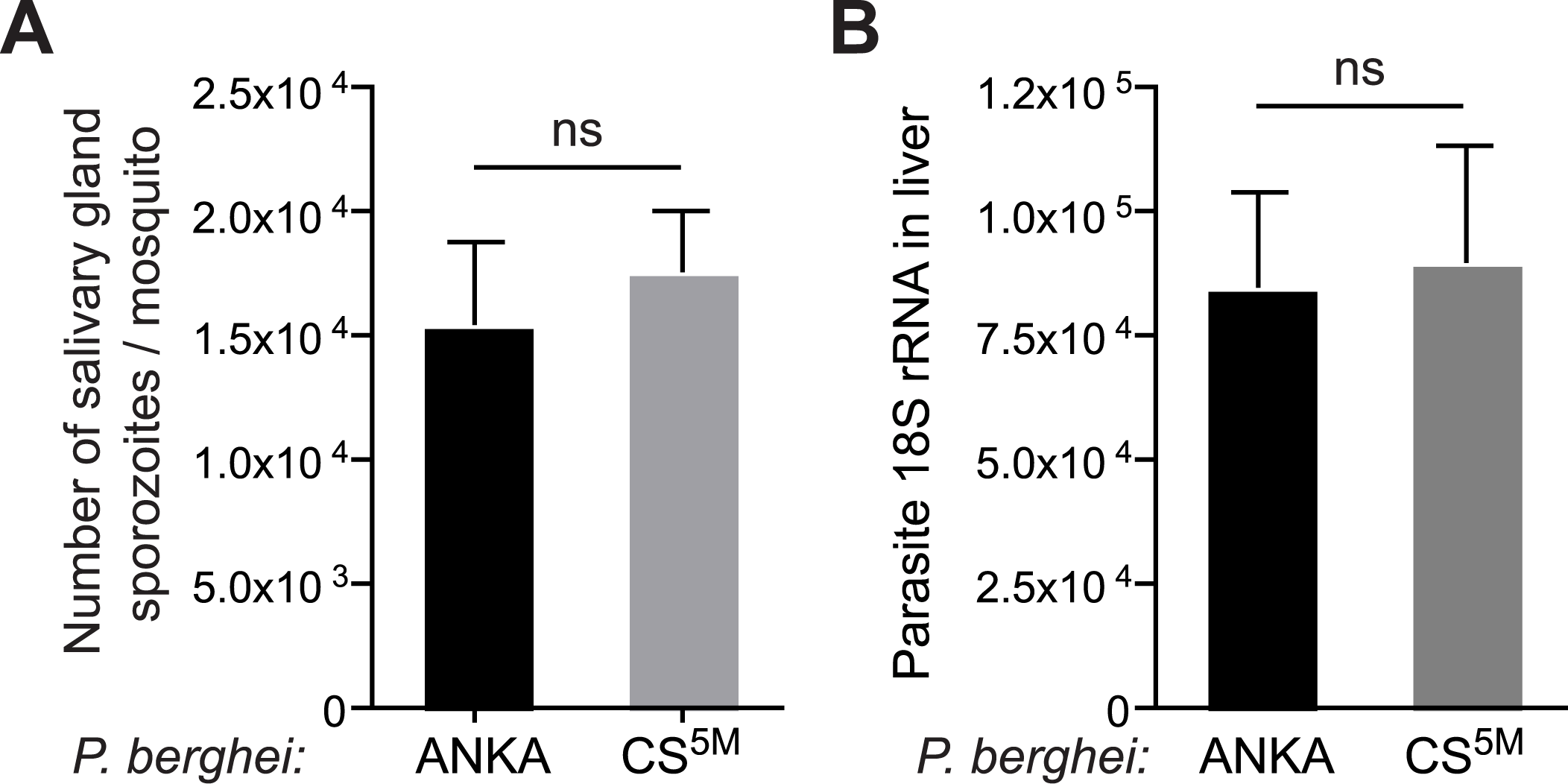

Supplement: Figure S1 — Infectivity P. berghei CS5M parasites in the mosquito and mouse. A. Salivary glands were dissected from mosquitoes 21 days after blood feeding with P. berghei ANKA or P. berghei CS5M and sporozoites extracted and counted. Results are based on 3 independent feedings per group with >10 mosquitoes dissected per feeding (mean ± SEM; ns = not significant). B. Parasite load in the livers of mice infected with P. berghei ANKA or P. berghei CS5M was assessed 40 hours after infection (mean ± SEM; n = 4, data from one of 2 similar experiments). (0.40 MB TIF) [file ppat.1001318.s001.tif]

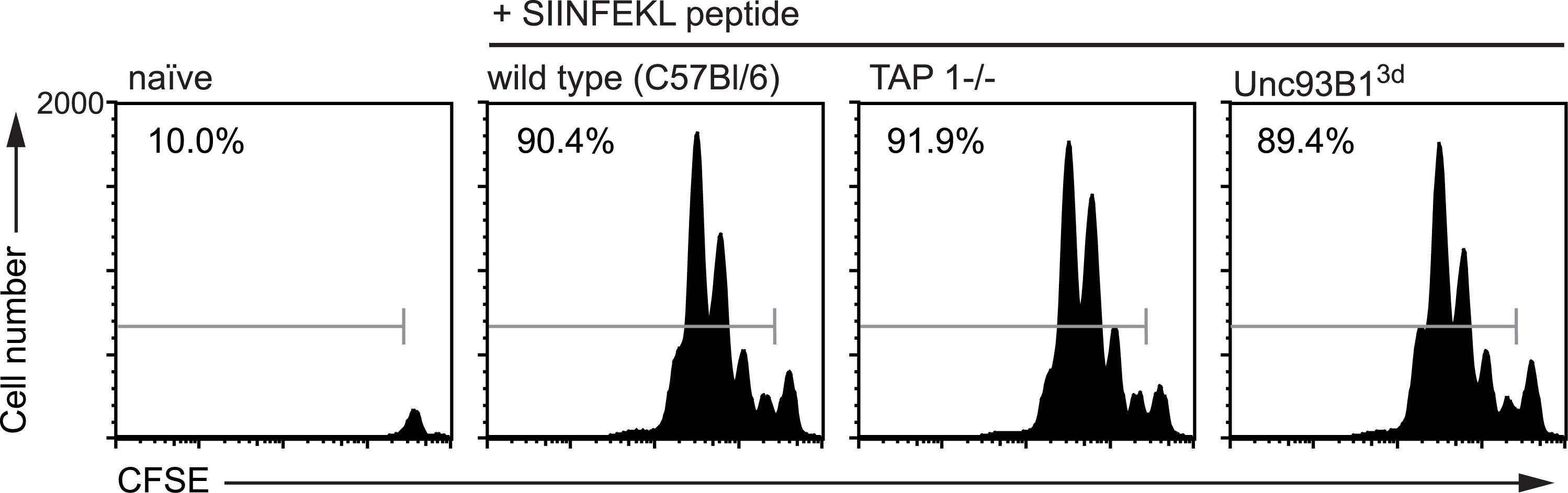

Supplement: Figure S2 — DCs from TAP-1 deficient and 3d mice can efficiently present exogenous peptide. CFSE profiles of SIINFEKL specific transgenic cells after incubation with spleen DCs isolated from C57Bl/6 (wild type), TAP-1 deficient or 3d mice that had been pulsed with 10μg/ml SIINFEKL peptide. As a control transgenic cells were also incubated with unpulsed DCs from naive wild type mice. Data are based on pooled DCs from 3 mice per group; values at top left are the percent of cells that have divided. Data is representative of two independent experiments per group. (0.33 MB TIF) [file ppat.1001318.s002.tif]

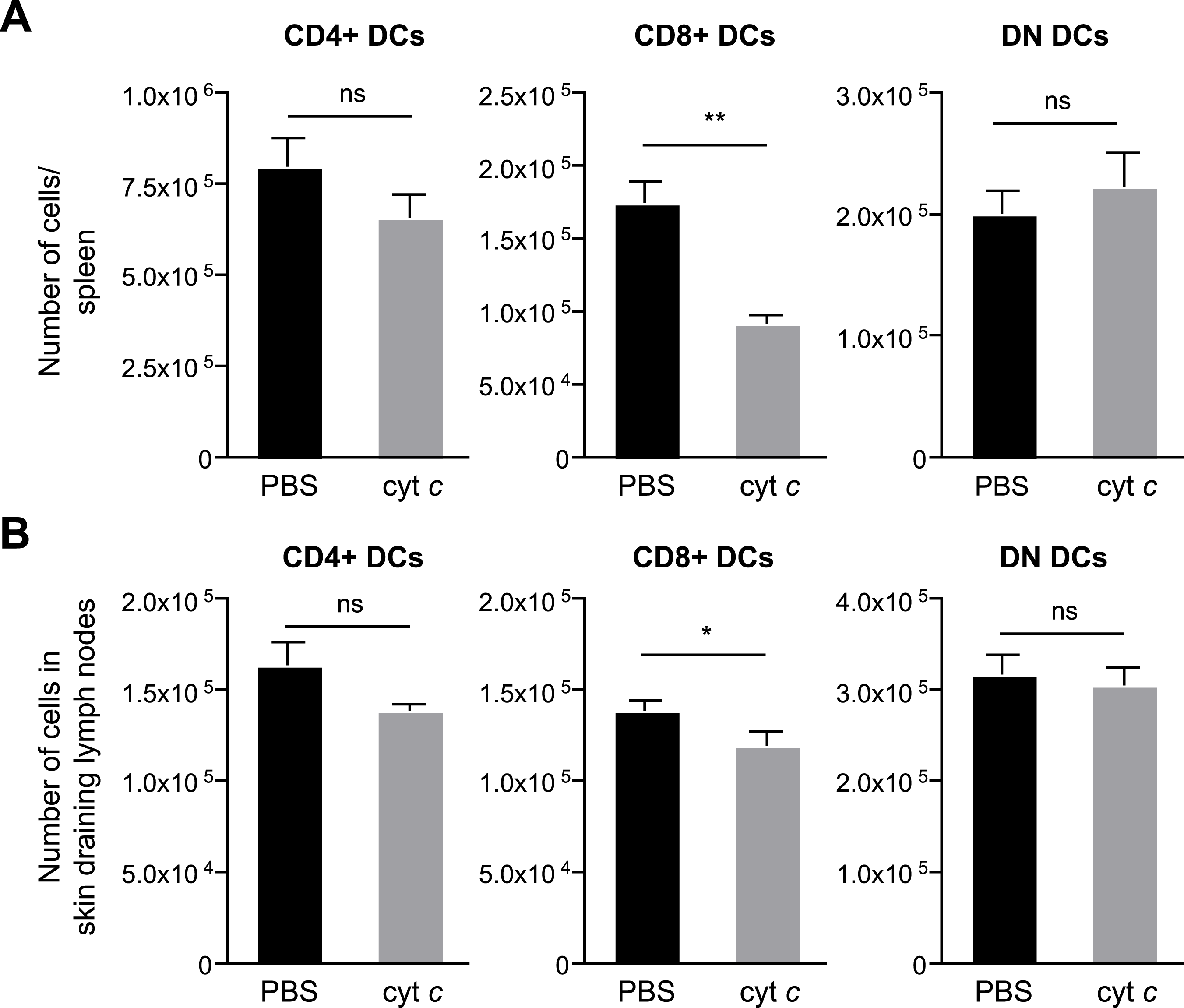

Supplement: Figure S3 — Cyt c treatment selectively ablates cross-presenting DC populations. Mice were treated with 15 mg horse cyt c in PBS or PBS alone, administered i.v. and 24 hours later the number of CD4+ DCs (CD4+, CD11c+, CD8−, CD3−) CD8+ DCs (CD8+, CD11c+, CD4−, CD3−) and double negative (DN) DCs (CD4−, CD8−. CD11c+, CD3−) was assessed in the spleen (A) and skin draining LNs (B) by FACs. (0.77 MB TIF) [file ppat.1001318.s003.tif]

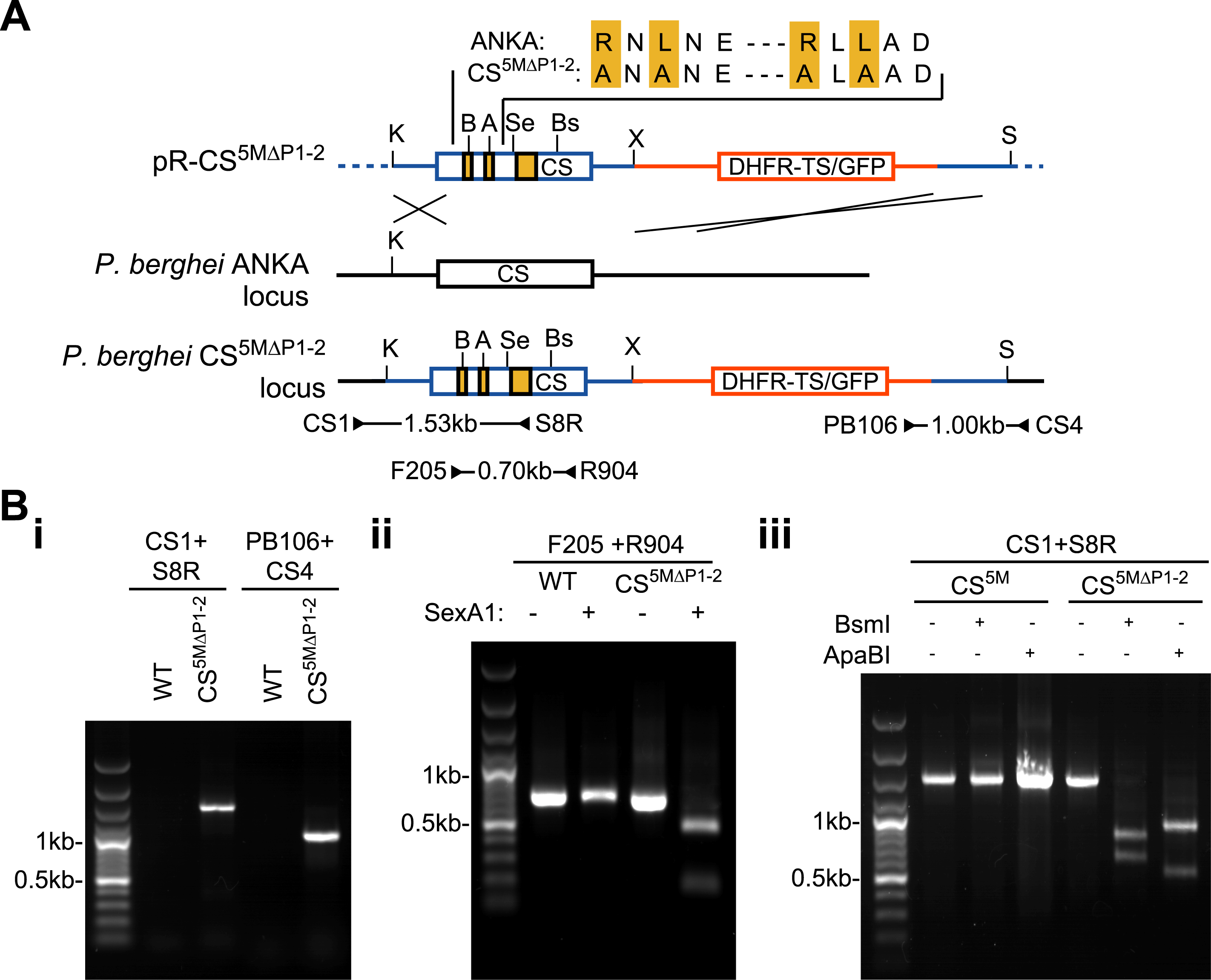

Supplement: Figure S4 — Generation of P. berghei CS5MΔP1–2 parasites. A. Scheme of the strategy used for gene targeting of the replacement CS5MΔP1–2 molecule. Open reading frames are represented by boxes, untranslated regions by solid lines, plasmid vectors sequences by dotted lines. Black represents wild type genomic sequences, blue represents homologous sequences in the targeting construct, red represents sequence associated with the selectable marker, and yellow represents mutations in the CS gene. Location of primers used for PCR verification of recombination is given below (primer sequences given in Table S1). Restriction sites are K - KpnI; B - BsmI; A - ApaBI; Se - SexAI; Bs - BsmF1; X - XhoI; S - SacI. B. Verification of clones - i. genomic DNA from cloned parasites was amplified with the primers CS1 and S8R (giving a 1526 bp product) to verify recombination at the 5′ end and the primers CS4 and PB106 (giving a 1001 bp product) to verify recombination at the 3′ end, genomic DNA from P. berghei ANKA was used as a control. ii To verify that parasite populations was clonal genomic DNA was amplified within the CS sequence with the primers F205 and R904 to give an 699 bp product in P. berghei ANKA and 696 bp product in P. berghei CS5MΔP1–2 (which has one codon less). The PCR product was then digested with SexA1 which cuts in the P. berghei CS5MΔP1–2 product, but not the P. berghei ANKA product, to yield fragments of 510 and 186 bp. iii To verify that the parasites carried mutations in the PEXEL domains, the CS1 and S8R PCR product was digested with the enzymes BsmI which cuts in the mutated Pexel1 motif to yield 618 and 845 bp fragments and ApaBI which cuts in the mutated Pexel2 motif to yield 581 and 945 bp fragments. The PCR product from the P. berghei CS5M parasite was used as a control. (1.46 MB TIF) [file ppat.1001318.s004.tif]
