# Supplementary material for: Dendritic Cells and Hepatocytes Use Distinct Pathways to Process Protective Antigen from Plasmodium in vivo
Source: PLoS Pathog. 2011 Mar 17;7(3):e1001318. doi: 10.1371/journal.ppat.1001318 (PMC3060173; doi:10.1371/journal.ppat.1001318)
Supplement: Table S1 — Oligonucleotides used in this study. (0.05 MB DOC) [file ppat.1001318.s005.doc]

**Table S1: Oligonucleotides used in this study**

| **Oligo** | **Sequence** | **Reference** |
| --- | --- | --- |
| CS4 | GGGCACGAATAGTAACTTATTTCG | Wang et al. (30) |
| PB106 | TGTGCATGCACATGCATGTA | Wang et al. (30) |
| CS1 | CTTTTTCACCCTCAAGTTGGG | Wang et al. (30) |
| S8R | TAGTTTTTCGAAATTGATGATAG | N/A |
| F205 | GCCGATGCTCCCGAAGGAAA | N/A |
| R699 | ATCTTCTGCTTTCTTATT | N/A |
| S8 insert F | CCAGGTGGTAATAACAATAACAAAAATAATAATAATGACGATTCTATCATCAA-  TTTCGAAAAACTACTAGAATTTGTTAAACAGATCAGGGACAGTATCACAG | N/A |
| S8 insert R | TCCTCTGTGATACTGTCCCTGATCTGTTTAACAAATTCTAGTAGTTTTTCGAAATT-GATGATAGAATCGTCATTATTATTATTTTTGTTATTGTTATTACCA | N/A |
| PEXEL1 F | GCATCCAAGCCCAAGCGAATGCAAACGAGCTATGTTACAATGAAGG | N/A |
| PEXEL1 R | CCTTCATTGTAACATAGCTCGTTTGCATTCGCTTGGGCTTGGATGC | N/A |
| PEXEL2 F | CAATCGAAATACAGTCAACGCATTAGCTGCCGATGCTCCCGAAGG | N/A |
| PEXEL2 R | CCTTCGGGAGCATCGGCAGCTAATGCGTTGACTGTATTTCGATTG | N/A |
